# Electroacupuncture at the Dazhui and Baihui acupoints and different frequencies (10 and 50 Hz) protects against apoptosis by up-regulating ERK1/2-mediated signaling in rats after global cerebral ischemia

**DOI:** 10.22038/IJBMS.2024.72279.15716

**Published:** 2024

**Authors:** Yueh-Ting Tsai, Chin-Yi Cheng

**Affiliations:** 1 School of Post-baccalaureate Chinese Medicine, College of Chinese Medicine, China Medical University, Taichung 40402, Taiwan; 2 Department of Traditional Chinese Medicine, Kuang Tien General Hospital, Taichung 43303, Taiwan; 3 Department of Chinese Medicine, Hui-Sheng Hospital, Taichung 42056, Taiwan; 4 Department of Chinese Medicine, China Medical University Hospital, Taichung 42056, Taiwan

**Keywords:** Apoptosis-inducing factor, Brain ischemia, Electroacupuncture, Hippocampus, MAP kinase signaling - system

## Abstract

**Objective(s):**

This study assessed the effects of electroacupuncture (EA) stimulation at different frequencies at the Dazhui and Baihui acupoints in the subacute phase after transient global cerebral ischemia (GCI).

**Materials and Methods:**

Rats were subjected to GCI for 25 min, followed by reperfusion for 7 days. EA at acupoints was performed at 10, 30, or 50 Hz, 1 day after reperfusion and then once daily for 6 consecutive days.

**Results:**

EA at acupoints at 10 and 50 Hz effectively down-regulated apoptosis in the hippocampal cornu ammonis 1(CA1) area and ameliorated memory deficits. Moreover, EA treatment at 10 and 50 Hz markedly increased phospho (p)-extracellular signal-regulated protein kinase 1/2 (ERK1/2), p-ERK1/2/neuronal nuclei (NeuN), p-cAMP response element-binding protein (CREB)/p-ERK1/2, B-cell lymphoma-2 (Bcl-2)/p-CREB, and X-linked inhibitor of apoptosis protein/NeuN expression levels and decreased Bcl-2 homologous antagonist/killer, second mitochondria-derived activator of caspase/direct inhibitor of apoptosis-binding protein with low pI, cytochrome c, cleaved caspase-3, and apoptosis-inducing factor expression levels. Furthermore, 10-Hz EA treatment effectively increased p-p38 mitogen-activated protein kinase (MAPK), p-p38 MAPK/NeuN, and p-CREB/p-p38 MAPK expression levels. Pretreatment with U0126 (ERK1/2 inhibitor) completely abrogated the effects of 10- and 50-Hz EA treatments on the aforementioned protein expression levels. Similarly, pretreatment with SB203580 (p38 MAPK inhibitor) completely abrogated the effects of 10-Hz treatment on the aforementioned protein expression levels.

**Conclusion:**

The effects of 10- and 50-Hz EA treatments on mitochondria-related apoptosis can be attributed to the activation of ERK1/2/p38 MAPK/CREB/Bcl-2- and ERK1/2/CREB/Bcl-2-mediated signaling, respectively, in the hippocampal CA1 area at 7 days after transient GCI.

## Introduction

Global cerebral ischemia (GCI) is a clinical outcome event in which the blood flow to the brain is reduced or stopped (1). Transient GCI triggers delayed neuronal death in the pyramidal neurons of the hippocampal cornu ammonis 1 (CA1) area, which plays a crucial role in learning and memory and is particularly vulnerable to ischemic insults (2). Thus, apoptosis-mediated delayed neuronal death in the hippocampal CA1 area could cause memory loss in the later stage following transient GCI (3).

Mitogen-activated protein kinases (MAPKs), including extracellular signal-regulated protein kinase 1/2 (ERK1/2), p38 MAPK, and c-Jun N-terminated kinase (JNK) are key regulators of apoptosis in response to cerebral ischemia–reperfusion (I/R) injury (4). In cerebral I/R injury, ERK1/2 plays a critical role in neuronal plasticity and survival and confers neuroprotection against apoptosis in the hippocampus (5, 6). The p38 MAPK pathway plays a dual role in apoptosis and cell survival following cerebral ischemia (7). During GCI, excessive production of reactive oxygen species (ROS) triggers the activation of the JNK signaling cascade, inducing apoptotic neuronal death in the hippocampus (8). A reciprocal relationship between ERK1/2- and p38 MAPK-mediated signaling has been found to regulate common downstream substrates in *in vitro* cell culture (9, 10) and *in vivo* GCI (11) models. Furthermore, in the homocysteine-N-methyl-D-aspartate receptor-induced neuronal cell injury model, p38 MAPK phosphorylation is dependent on ERK1/2 activity, and p38 MAPK is a downstream mediator of ERK1/2-related signaling (12). The cAMP response element-binding protein (CREB), a downstream target of ERK1/2 and p38 MAPK, is a nuclear transcription factor, that induces the expression of anti-apoptotic genes, such as B-cell lymphoma-2 (*Bcl-2*) and Bcl-extra-large (*Bcl-xL*), and confers neuroprotection against cerebral ischemic injury (13). In transient GCI, CREB activation protects hippocampal neurons against I/R injury and is associated with neuronal survival, memory recovery, and synaptic plasticity (5). Cerebral I/R triggers oxidative stress in response to ROS accumulation in the ischemic area. Subsequently, the mitochondria are damaged by oxidative stress and the insertion and oligomerization of the pro-apoptotic factors Bcl-2-associated x protein (Bax) and Bcl-2 homologous antagonist/killer (Bak), leading to mitochondrial outer membrane (MOM) permeabilization (14). Thus, mitochondria-associated pro-apoptotic factors, such as cytochrome c (cyt c), second mitochondria-derived activator of caspase/direct inhibitor of apoptosis-binding protein with low pI (Smac/DIABLO), and apoptosis-inducing factor (AIF) are released into the cytosol (15). The released cyt c binds to apoptotic protease activating factor-1 (Apaf-1) and pro-caspase-9 to form an apoptosome, which then initiates caspase-3-mediated apoptosis in the ischemic penumbra (16). Smac/DIABLO released from the mitochondria into the cytosol can bind to X-linked inhibitor of apoptosis protein (XIAP), a member of the inhibitor of apoptosis protein family, and eventually trigger caspase activation. Moreover, AIF, a mitochondrial intermembrane protein, translocates from the mitochondria into the nucleus, leading to caspase-independent apoptosis (17). 

Acupuncture stimulation at acupoints is a critical component of traditional Chinese medicine. Electroacupuncture (EA) is based on traditional acupuncture combined with modern electrical stimulation, and it promotes the effects of acupuncture treatments (18). Liu *et al*. reported that EA (1/20 Hz, 1 mA) stimulation at the Baihui (GV20) and Shenting (GV24) acupoints exerts beneficial effects on apoptosis by suppressing p38 MAPK- and JNK-mediated signaling and enhancing ERK1/2-mediated signaling in the ischemic penumbra in the subacute phase after transient focal cerebral ischemia (19). Moreover, EA (5 and 50 Hz, 1–2 mA) stimulation at the Dazhui (GV14) and GV20 acupoints can protect against memory impairment by up-regulating the expression of brain-derived neurotrophic factor (BDNF) and nerve growth factor in the hippocampal CA3 region at 14 days after transient middle cerebral artery occlusion (MCAO) (20). Our previous studies have revealed that EA (5 Hz, 2.7–3.0 mA) stimulation at the GV14 and GV20 acupoints exerts neuroprotective effects against cerebral I/R-induced apoptosis by up-regulating BDNF/ERK1/2-mediated signaling (21) and down-regulating p38 MAPK/nuclear factor-κB/cleaved caspase-3-mediated signaling (22) in the peri-infarct region during the subacute phase of cerebral ischemia. Moreover, EA stimulation at the Fengfu (GV16) and GV20 acupoints at 5 and 25 Hz confers neuroprotective effects against cerebral infarction, partly through the activation of p38 MAPK/CREB-mediated anti-apoptotic signaling in the cortical penumbra during the subacte phase after cerebral I/R injury (23). Based on the aforementioned findings, we conclude that EA stimulation at the GV14 and GV20 acupoints (hereafter, EA at acupoints) at different frequencies (5–50 Hz) exhibits neuroprotective effects against cerebral I/R injury by modulating MAPK-mediated anti-apoptotic signaling in the ischemic region during the subacute phase after MCAO. However, the effects of EA at acupoints at different frequencies on hippocampal apoptosis and MAPK-related signaling during GCI still need to be clarified.

Hence, in this study, we assessed the effects of EA at acupoints, at 10, 30, or 50 Hz, performed after 7 days of reperfusion after 25 min of GCI, and examined the involvement of MAPK-mediated anti-apoptotic signaling in the hippocampus. 

## Materials and Methods

***Experimental animals***

Male Sprague Dawley rats (BioLasco Taiwan Co., Ltd., Yi-Lan Country) weighing 300–330 g were purchased and housed in cages under controlled environmental conditions at a temperature of 22 ± 2 °C and relative humidity of 55% under a 12-hr light/dark cycle. All experimental procedures in this study were performed after obtaining the ethical approval of the Institutional Animal Care and Use Committee of China Medical University (permit number: CMUIACUC-2020-048).

***Transient GCI model***

 A rat model of transient GCI was established using the four-vessel occlusion (4-VO) method, as described previously (24). In brief, the rats were anesthetized through isoflurane inhalation (5% for induction and 1.5% for maintenance), and a 3-cm posterior midline incision was then conducted to explore the alar foramen of the first cervical vertebra. After the completion of permanent vertebral artery occlusion using electrocautery, the incisions were sutured, and the rats were placed in individual cages. On the second day, another 3-cm incision was performed in the anterior midline neck to explore bilateral common carotid arteries (CCAs), which were occluded for 25 min using arterial clamps and subsequently reopened after releasing the arterial clamps. The rats that had lost righting and corneal reflexes and exhibited dilated pupils during the ischemic period were considered successful 4-VO model rats and were included for further study. 

***EA stimulation method***

 Each rat was placed in a plastic chamber and underwent isoflurane (5%) anesthesia. Subsequently, the anesthetized rat was removed from the chamber with anesthesia maintained by the delivery of isoflurane (1.5%) through a face mask. EA stimulation was performed at GV14 (below the spinous process of the seventh cervical vertebra) and GV20 (midpoint of the parietal bone) acupoints by inserting acupuncture needles (1.5 cm, Han Bor International Co., Ltd., Taiwan) vertically at a depth of 5 mm and forward at a depth of 4 mm, respectively (receiving EA at acupoints). The rats that received EA stimulation in bilateral costal regions were classified as nonacupoint stimulation rats (receiving EA at nonacupoints). An EA apparatus (Trio 300, ITO Co., Saitama, Japan) was used to provide EA at acupoints or nonacupoints for 25 min each day for 6 consecutive days. The stimulation parameters were as follows: 10, 30, or 50 Hz constant wave; 2.7–3.0 mA; and 150-µs pulse width. 

***Passive avoidance task***

A passive avoidance task was performed following modified previously described procedures (25). In brief, the passive avoidance test was performed in a two-compartment box with a bright room and a dark room (25 × 20 × 17 cm^3^ each). Each memory test consisted of training and retention programs. The training program was conducted 1 hr before surgery. During this process, the rats were individually placed in the bright room, and the guillotine door was opened. When a rat moved from the bright room to the dark room, the door was immediately closed, and the rat received brief electric foot stimulation (50 Hz, 0.55 mA, 3 sec). The training program was stopped when the rat did not move to the dark room after 120 sec. The retention program was conducted without electric foot stimulation at 7 days after GCI. The time taken for the rat to move to the darkroom (step-through latency [STL]) was recoded; the maximum period was recorded as 300 sec.

**Experiment A**

***Grouping***

The rats were randomly divided into nine groups (n = 5 per group): Sham, Model, Non-acup, 10 Hz, 30 Hz, 50 Hz, U+10 Hz, U+50 Hz, and SB+10 Hz groups. A day after transient GCI, the rats in the 10 Hz, 30 Hz, and 50 Hz groups received the first EA stimulation at acupoints at 10, 30, and 50 Hz, respectively, for 25 min. Subsequently, they received EA at acupoints once daily for 6 consecutive days. After completion of the passive avoidance test after 7 days of reperfusion, the rats underwent CO_2_ euthanasia and their brains were quickly removed. The rats in the Non-acup group were subjected to identical procedures as the rats in the 50 HZ group; however, they received EA stimulation at nonacupoints. The rats in the U+10 Hz and U+50 Hz groups were subjected to identical procedures as those conducted for the rats in the 10 Hz and 50 Hz groups, respectively. However, they were intracerebroventricularly (ICV) injected with U0126, a selective inhibitor of ERK1/2, 20 min before CCA ligation. Similarly, the rats in the SB+10 Hz groups were subjected to identical procedures as those conducted in the 10 Hz groups. However, they were ICV injected with SB203580, a specific inhibitor of p38 MAPK, 20 min before CCA ligation. The rats in the Model group were subjected to identical procedures as those in the 50 Hz group; however, they did not receive electrical stimulation. The rats in the Sham group were subjected to identical procedures as those in the Model group; however, in the sham group, bilateral CCAs were not occluded. 

***Terminal deoxynucleotidyl transferase dUTP nick-end labeling assay***

Serial coronal sections were stained using the terminal deoxynucleotidyl transferase dUTP nick-end labeling (TUNEL) method for detecting apoptotic cells. The coronal brain sections were incubated with proteinase K solution (QIA33, Sigma-Aldrich, Darmstadt, Germany) at a concentration of 20 µg/ml at room temperature (RT) for 20 min, and the subsequent procedures were performed as described previously (21). TUNEL-positive cells were evaluated within the selected hippocampal CA1 area (three 400-fold magnification fields) under a light microscope.

**Experiment B**

***Grouping***

The rats were randomly divided into six groups (n = 5 per group): Sham, Model, Non-acup, 10 Hz, 30 Hz, and 50 Hz groups. The procedures performed in these groups were identical to those performed in Experiment A.

***Western blot analysis***

After 7 days of reperfusion, the rats were euthanized, and their brains were immediately harvested and stored at −80 °C. Subsequently, the hippocampal tissues on both sides were carefully separated and quickly homogenized on ice. The protein contents in the samples were determined using the Bio-Rad protein assay. Equal amounts (15 μg) of protein samples were loaded into wells of 10% sodium dodecyl sulfate–polyacrylamide gel and subjected to electrophoresis, as described previously (26). After electrophoretic transfer, the separated proteins on the nitrocellulose membranes were incubated with the primary antibodies (listed in Table 1) at 4 °C overnight and subsequently incubated with goat anti-rabbit IgG (H+L) (1:5000, Jackson/AB_2313567) or goat anti-mouse IgG (H+L) (1:5000, Jackson/AB_10015289) secondary antibody at RT for 1 hr. Densitometric analyses were performed using image processing software (ImageJ 1.47v, National Institutes of Health).

**Experiment C**

***Grouping***

The rats were randomly divided into seven groups (n = 4 per group): D+Sham, D+Model, D+10 Hz, D+50 Hz, U+10 Hz, U+50 Hz, and SB+10 Hz groups. The rats in the D+Sham, D+Model, D+10 Hz, and D+50 Hz groups were subjected to identical procedures as those conducted for the Sham, Model, 10 Hz, and 50 Hz groups, respectively. However, they were ICV injected with 1% dimethyl sulfoxide (DMSO) 20 min before CCA ligation. The procedures performed in the U+10 Hz, U+50 Hz, and SB+10 Hz groups were identical to those performed in Experiment A. Intracerebroventricular injections of 1% DMSO in the D+Sham, D+Model, D+10 Hz, and D+50 Hz groups could thereby mask the effects of the solvent (1% DMSO) on target proteins in the hippocampus in the U+10 Hz, U+50 Hz, and SB+10 Hz groups.

***Intracerebroventricular injection of U0126, SB203580, or 1% DMSO***

The rats were anesthetized, and two 1-mm-diameter burr holes were symmetrically made 2 mm lateral and 3.5 mm posterior to the bregma of the skull. To suppress the activation of ERK1/2 and p38 MAPK in the hippocampus, intracerebroventricular injections of U0126 and SB203580 were performed, respectively. The rats were ICV injected with 6 μl of U0126 (0.67 μg/μl U0126 dissolved in 1% DMSO, CAS109511-58-2, Sigma-Aldrich), 10 μl of SB203580 (2 mM SB203580 dissolved in 1%DMSO, #S1076, Selleckchem.com), or 10 μl of 1% DMSO solution through each burr hole (3.5 mm depth) using a 10-µl Hamilton syringe (Hamilton Company, Reno, NV, USA).

***Western blot analysis***

After 7 days of reperfusion, the rats were sacrificed, and their brains were quickly removed for detecting phospho (p)-ERK1/2, ERK1/2, p-p38 MAPK, and p38 MAPK (Table 1) expression levels through western blot analysis. The analysis was conducted using identical procedures to those performed in Experiment B. 

**Experiment D**

***Grouping***

The rats were randomly divided into nine groups (n = 5 per group): Sham, Model, Non-acup, 10 Hz, 30 Hz, 50 Hz, U+10 Hz, U+50 Hz, and SB+10 Hz groups. The procedures performed in these groups were identical to those conducted in Experiment A. 

***Immunofluorescence double staining***

The coronal brain sections adjacent to those used in TUNEL staining were subjected to immunofluorescence (IF) double staining. The brain sections were postfixed with 4% paraformaldehyde (PFA) at RT for 15 min and incubated with the primary antibodies listed in Table 1 at 4 °C overnight and subsequently incubated with appropriate anti-rabbit and anti-mouse IgG secondary antibodies at 37 °C for 1.5 hr, as described previously (27). The immunopositive cells in the selected hippocampal CA1 area were measured in each of the three 400-fold magnification fields under a fluorescence microscope (CKX53, Olympus, Tokyo, Japan).

***Immunohistochemical staining***

The coronal brain sections were postfixed in 4% PFA at RT for 15 min and then incubated with the primary antibodies listed in Table 1 at 4 ºC overnight. Subsequent immunohistochemical (IHC) staining was performed as described previously (27). The immunopositive cells in the selected hippocampal CA1 area were measured in each of the three 400-fold magnification fields under a light microscope. The brain coronal sections obtained from the Model group were stained without the primary antibodies and were considered negative controls.

***Statistical analysis***

All numerical data obtained from the passive avoidance task, TUNEL assay, western blot analysis, IF assay, and IHC analysis among the experimental groups followed a normal distribution and were compared using one-way analysis of variance, followed by the Bonferroni *post hoc* test. All data are expressed as mean ± standard deviation (SD). A *P*-value of < 0.05 indicated statistical significance. All data were analyzed using SPSS 13.0 software (SPSS, Inc., Chicago, IL, USA).

## Results

***Effects of 10- and 50-Hz treatments on the expression of TUNEL, p-ERK1/2/neuronal nuclei, and p-p38 MAPK/neuronal nuclei and memory deficits***

**Figure 1:**
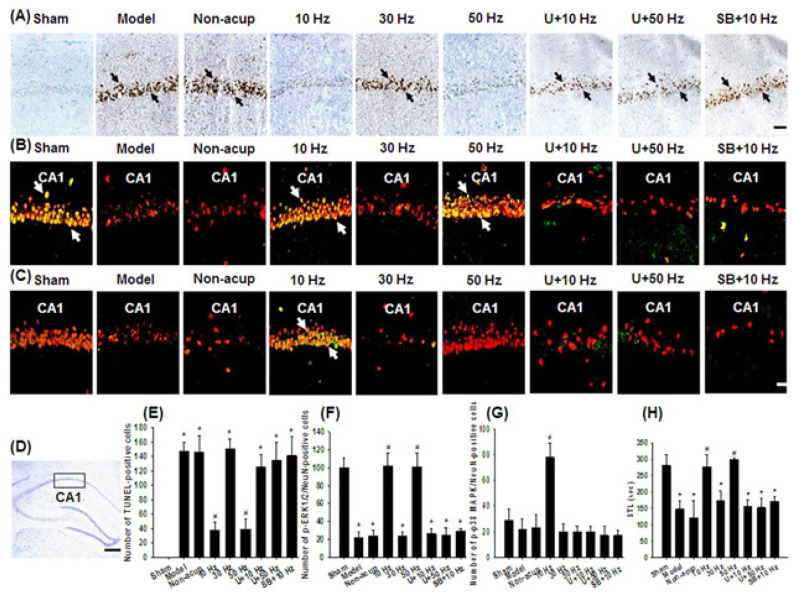
Effects of 10- and 50-Hz treatments on TUNEL, p-ERK1/2/NeuN, and p-p38 MAPK/NeuN expression in the hippocampal CA1 area and memory deficits in the experimental groups at 7 days after transient GCI

The numbers of immunopositive cells were evaluated in the selected hippocampal CA1 area (Figure 1D). The number of TUNEL-positive cells in the hippocampal CA1 area was significantly higher in the Model, Non-acup, 30 Hz, U+10 Hz, U+50 Hz, and SB+10 Hz groups than in the Sham group (all *P*<0.05) and was significantly lower in the 10 and 50 Hz groups than in the Model group (both *P*<0.05; Figures 1A and 1E). The number of p-ERK1/2/neuronal nuclei (NeuN)-positive cells in the hippocampal CA1 area was significantly lower in the Model, Non-acup, 30 Hz, U+10 Hz, U+50 Hz, and SB+10 Hz groups than in the Sham group (all *P*<0.05) and was significantly higher in the 10 and 50 Hz groups than in the Model group (both *P*<0.05; Figures 1B and 1F). However, the aforementioned immunopositive cells did not differ markedly among the Model, Non-acup, 30 Hz, U+10 Hz, U+50 Hz, and SB+10 Hz groups (all *P*>0.05). The number of p-p38 MAPK/NeuN-positive cells in the hippocampal CA1 area was significantly higher in the 10 Hz group than in the Model group (*P*<0.05; Figures 1C and 1G). However, p-p38 MAPK/NeuN double-labeled cells did not differ markedly among the Sham, Model, Non-acup, 30 Hz, 50 Hz, U+10 Hz, U+50 Hz, and SB+10 Hz groups (all *P*>0.05). STL was significantly lower in the Model, Non-acup, 30 Hz, U+10 Hz, U+50 Hz, and SB+10 Hz groups than in the Sham group (all *P*<0.05) and was significantly higher in the 10 and 50 Hz groups than in the Model group (both *P*<0.05; Figure 1H). However, the number of STLs did not differ markedly among the Model, Non-acup, 30 Hz, U+10 Hz, U+50 Hz, and SB+10 Hz groups (all *P*>0.05).

***Effects of 10- and 50-Hz treatments on the expression of p-ERK1/2, ERK1/2, p-p38 MAPK, p38 MAPK, p-JNK, JNK, p-Akt, and Akt***

**Figure 2:**
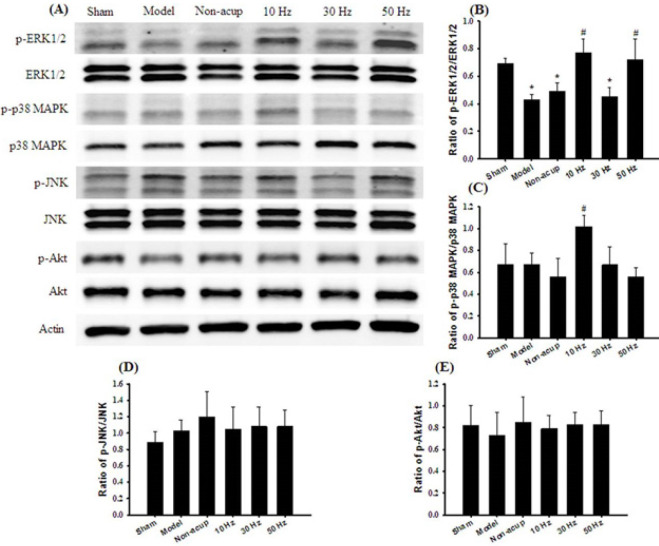
Effects of 10- and 50-Hz treatments on MAPKs, p-Akt, and Akt expression in the hippocampus in the experimental groups at 7 days after transient GCI

The ratio of p-ERK1/2 to ERK1/2 expression in the hippocampus was significantly lower in the Model (0.6-fold), Non-acup (0.7-fold), and 30 Hz (0.7-fold) groups than in the Sham group (all *P*<0.05) and was significantly higher in the 10 Hz (1.8-fold) and 50 Hz (1.7-fold) groups than in the Model group (both *P*<0.05; Figures 2A and 2B). The ratio of p-ERK1/2 to ERK1/2 expression did not differ markedly among the Model, Non-acup, and 30 Hz groups (all *P*>0.05). In addition, the ratio of p-p38 MAPK to p38 MAPK expression in the hippocampus was significantly higher in the 10 Hz group (1.5-fold) than in the Model group (*P*<0.05; Figures 2A and 2C). The ratio of p-p38 MAPK to p38 MAPK expression did not differ markedly among the Sham, Model, Non-acup, 30 Hz, and 50 Hz groups (all *P*>0.05). Similarly, the ratios of p-JNK to JNK expression and p-Akt to Akt expression did not differ markedly among the experimental groups (all *P*>0.05; Figures 2A, 2D, and 2E). 

***Effects of D+10 Hz and D+50 Hz treatments on the ratios of p-ERK1/2 to ERK1/2 and p-p38 MAPK to p38 MAPK expression***

**Figure 3:**
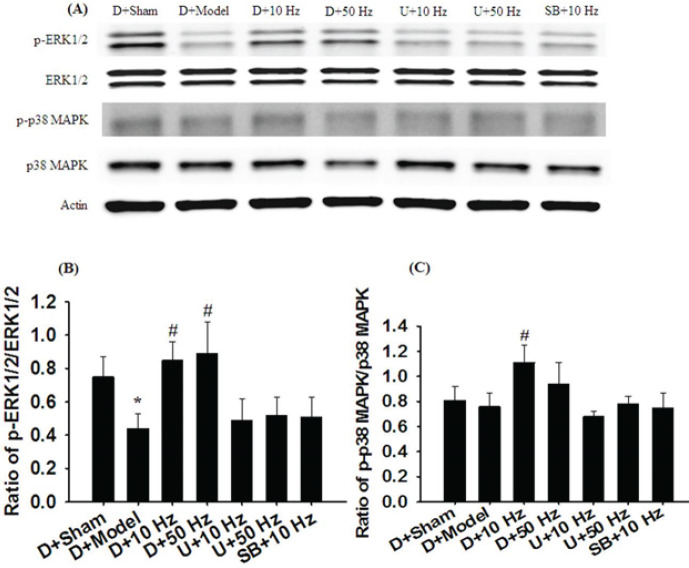
Effects of D+10-Hz and D+50-Hz treatments on p-ERK1/2, ERK1/2, p-p38 MAPK, and p38 MAPK expression in the hippocampus in the experimental groups at 7 days after transient GCI

The ratio of p-ERK1/2 to ERK1/2 expression in the hippocampus was significantly lower in the D+Model (0.6-fold) group than in the D+Sham group (all *P*<0.05) and was significantly higher in the D+10 Hz (1.9-fold) and D+50 Hz (2.0-fold) groups than in the D+Model group (both *P*<0.05; Figures 3A and 3B). However, the ratio of p-ERK1/2 to ERK1/2 expression did not differ markedly among the D+Model, U+10 Hz, U+50 Hz, and SB+10 Hz groups (all *P*>0.05). Moreover, the ratio of p-p38 MAPK to p38 MAPK expression in the hippocampus was significantly higher in the D+10 Hz group (1.5-fold) than in the D+Model group (*P*<0.05; Figures 3A and 3C). However, the ratio of p-p38 MAPK to p38 MAPK expression did not differ markedly among the D+Sham, D+Model, D+50 Hz, U+10 Hz, U+50 Hz, and SB+10 Hz groups (all *P*>0.05). Moreover, the expression levels of p-ERK1/2/ERK1/2 and p-p38 MAPK/p38 MAPK in the D+Sham, D+Model, D+10 Hz, and D+50 Hz groups were similar to those in the Sham, Model, 10 Hz, and 50 Hz groups, respectively.

***Effects of 10- and 50-Hz treatments on the expression of p-CREB/p-ERK1/2, p-CREB/p-p38 MAPK, and Bcl-2/p-CREB double-labeled cells***

**Figure 4:**
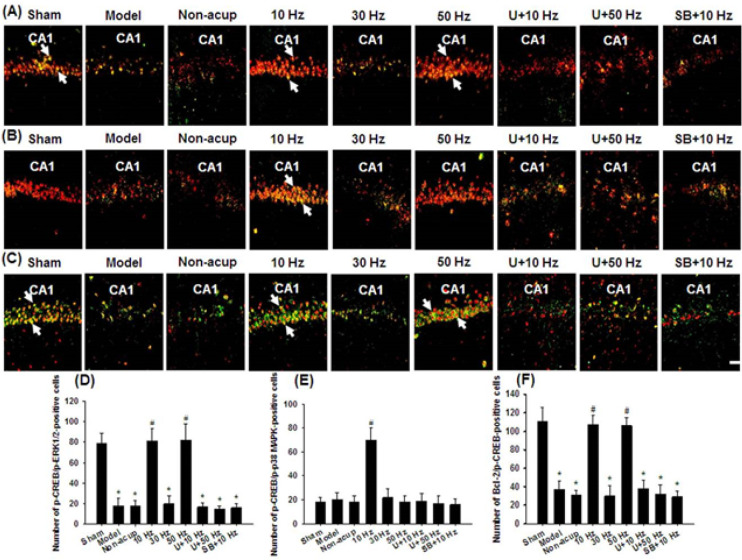
Effects of 10- and 50-Hz treatments on p-CREB/p-ERK1/2, p-CREB/p-p38 MAPK, and Bcl-2/p-CREB expression in the hippocampal CA1 area in the experimental groups at 7 days after transient GCI

The numbers of p-CREB/p-ERK1/2- and Bcl-2/p-CREB-positive cells in the hippocampal CA1 area were significantly lower in the Model, Non-acup, 30 Hz, U+10 Hz, U+50 Hz, and SB+10 Hz groups than in the Sham group (all *P*<0.05) and were significantly higher in the 10 and 50 Hz groups than in the Model group (all *P*<0.05; Figures 4A, 4C, 4D, and 4F). However, the aforementioned double-labeled cells did not differ markedly among the Model, Non-acup, 30 Hz, U+10 Hz, U+50 Hz, and SB+10 Hz groups (all *P*>0.05). The number of p-CREB/p-p38 MAPK-positive cells in the hippocampal CA1 area was significantly higher in the 10 Hz group than in the Model group (*P*<0.05; Figures 4B and 4E). However, p-CREB/p-p38 MAPK double-labeled cells did not differ markedly among the Sham, Model, Non-acup, 30 Hz, 50 Hz, U+10 Hz, U+50 Hz, and SB+10 Hz groups (all *P*>0.05).

***Effects of 10- and 50-Hz treatments on the expression of Bak-, Smac/DIABLO-, cyt c-, XIAP/NeuN-, cleaved caspase-3-, and AIF-positive cells***

**Figure 5:**
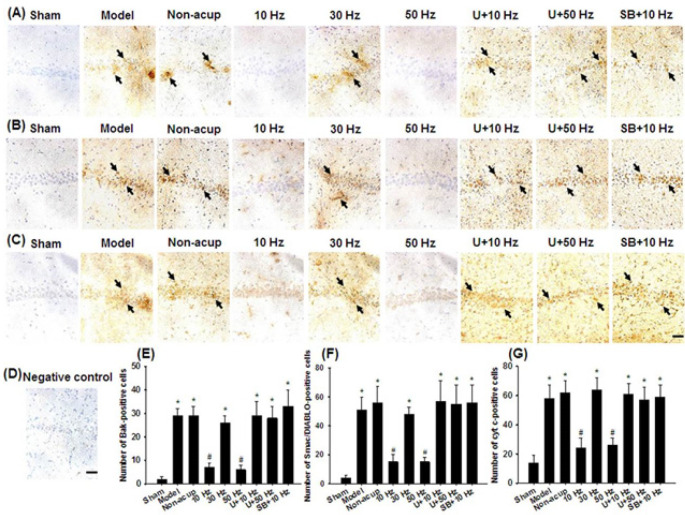
Effects of 10- and 50-Hz treatments on Bak, Smac/DIABLO, and cyt c expression in the hippocampal CA1 area in the experimental groups at 7 days after transient GCI

**Figure 6:**
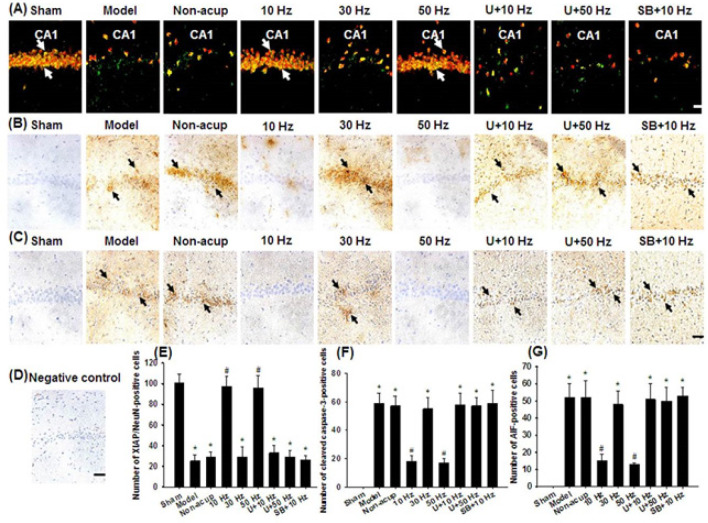
Effects of 10- and 50-Hz treatments on XIAP/NeuN, cleaved caspase-3, and AIF expression in the hippocampal CA1 area in the experimental groups at 7 days after transient GCI

The numbers of Bak-, Smac/DIABLO, cyt c-, cleaved caspase-3, and AIF*-*positive cells in the hippocampal CA1 area were significantly higher in the Model, Non-acup, 30 Hz, U+10 Hz, U+50 Hz, and SB+10 Hz groups than in the Sham group (all *P*<0.05) and were significantly lower in the 10 and 50 Hz groups than in the Model group (all *P*<0.05; Figures 5A–5G, 6B–6D, 6F, and 6G). By contrast, the number of XIAP/NeuN-positive cells in the hippocampal CA1 area was significantly lower in the Model, Non-acup, 30 Hz, U+10 Hz, U+50 Hz, and SB+10 Hz groups than in the Sham group (all *P*<0.05) and was significantly higher in the 10 and 50 Hz groups than in the Model group (both *P*<0.05; Figures 6A and 6E). However, the aforementioned immunopositive cells did not differ markedly among the Model, Non-acup, 30 Hz, U+10 Hz, U+50 Hz, and SB+10 Hz groups (all *P*>0.05).

## Discussion

Generally, transient GCI occurs because of carotid artery occlusion or systemic hypoperfusion, which leads to reduced blood flow to the brain tissue (28). Pyramidal neurons in the hippocampal CA1 area initiate apoptotic death 3–7 days after global cerebral I/R injury, resulting in memory impairment in the subsequent period (29, 30). Zhu *et al*. reported that EA at acupoints (GV14 and GV20) at a frequency of 4 Hz can protect against neuronal apoptosis in the hippocampal CA1 area and attenuate memory impairment; these effects are partly mediated by the suppression of p53/Noxa-related signaling in the chronic phase after GCI (31). Our previous study has revealed that EA at acupoints at a frequency of 5 Hz confers neuroprotective effects against cerebral I/R-induced apoptosis by up-regulating ERK1/2-mediated signaling in the penumbra regions during the subacute phase after MCAO (21). Moreover, EA stimulation at frequencies between 4 and 20 Hz confers beneficial effects against apoptotic cell death in the ischemic area during the subacute phase of ischemic stroke (32). In this study, we assessed the relationship between different frequencies and the effects of EA at acupoints on neuronal apoptosis in the hippocampal CA1 area following transient GCI. Our results revealed that 25 min of 4-VO ischemia (Model group) elicited severe neuronal apoptotic death in the hippocampal CA1 area at 7 days after reperfusion. However, EA at acupoints at frequencies of 10 Hz (10 Hz group) and 50 Hz (50 Hz group) once daily for 6 consecutive days effectively rescued hippocampal CA1 neuronal apoptotic death and improved memory deficits in the subacute phase after transient GCI. By contrast, EA at nonacupoints at 50 Hz (Non-acup group) and EA at acupoints at 30 Hz (30 Hz group) did not rescue hippocampal CA1 pyramidal neuron death induced by global cerebral I/R injury. Hence, we infer that EA at acupoints at 10 and 50 Hz, but not at 30 Hz, effectively ameliorates memory impairment, partly through the suppression of apoptosis in the hippocampal CA1 area at 7 days after transient GCI. 

MAPKs, including ERK1/2, p38 MAPK, and JNK, are a family of serine/threonine kinases that regulate intracellular apoptotic signaling in the hippocampus in response to I/R injury following transient GCI (11). Activation of the ERK1/2 pathway is associated with the regulation of cell death and survival in the ischemic area during cerebral ischemia (5). Activation of ERK1/2-related signaling can protect neurons from cyt c/caspase-3-induced apoptosis in the hippocampal CA1 area during the subacute (4) and chronic (33) phases after GCI. p38 MAPK is a stress-activated protein kinase that plays a dual role in the induction of neuronal apoptosis and the development of neuronal tolerance in the hippocampus in various GCI models (34). Furthermore, during cerebral ischemia, simultaneous activation of ERK1/2 and p38 MAPK in the hippocampus contributes to the inhibition of apoptosis after I/R injury. This mutual crosstalk suggests a consistently positive relationship (11, 13). Akt signaling plays a crucial role in the regulation of cell survival, proliferation, and differentiation (35). Activation of Akt promotes neuronal survival by down-regulating cyt c/caspase-3-mediated apoptotic signaling in the ischemic area during the subacute phase after cerebral ischemia (36). Our western blot and IF results revealed that p-ERK1/2 and p-ERK1/2/NeuN (a specific neuronal marker) expression was markedly down-regulated in the hippocampal CA1 area after global cerebral ischemic injury. Although 10- and 50-Hz treatments effectively up-regulated p-ERK1/2 and p-ERK1/2/NeuN expression, they did not affect p-JNK and p-Akt expression in the hippocampal CA1 area during the subacute phase after reperfusion. Moreover, 10-Hz treatment effectively up-regulated p-p38 MAPK and p-p38 MAPK/NeuN expression in the hippocampal CA1 area. These results suggest that 10- and 50-Hz treatments exert neuroprotective effects against neuronal apoptosis, possibly through mutual interaction between ERK1/2 and p38 MAPK signaling and the activation of ERK1/2 signaling, respectively, in the hippocampal CA1 area at 7 days after transient GCI.

In cerebral I/R injury, activated ERK1/2 and p38 MAPK lead to the phosphorylation of the common downstream target CREB in the ischemic region (37, 38). CREB is a crucial transcription factor involved in neuronal survival, memory formation, and synaptic plasticity in the hippocampus. Hence, CREB activation exerts neuroprotective effects against I/R injury in various models of cerebral ischemia (5). p-CREB located in the nucleus binds to cAMP response element-containing promoters and induces the expression of its target genes, including *Bcl-2* and *BDNF* genes (37). Bcl-2 family members include anti-apoptotic (e.g., Bcl-2 and Bcl-xL) and pro-apoptotic (e.g., Bax and Bak) proteins. The anti-apoptotic proteins promote neuronal survival by suppressing pro-apoptotic protein expression and preserving MOM integrity in the ischemic area after cerebral ischemia. By contrast, the pro-apoptotic proteins trigger ischemia-induced neuronal death by inducing the release of apoptogenic factors (such as cyt c, Smac/DIABLO, and AIF) from the mitochondria into the cytosol and subsequently activating the caspase-dependent and -independent apoptotic cascades following cerebral I/R injury (14, 37). Increased ratios of anti-apoptotic to pro-apoptotic Bcl-2 family proteins exert neuroprotective effects against I/R-induced apoptosis in the hippocampus during GCI (1, 39). Moreover, activation of ERK1/2/CREB-related signaling protects against apoptosis in the hippocampus and attenuates memory impairment in the subacute phase after transient GCI (5, 6). Furthermore, activation of the p38 MAPK/CREB/Bcl-2 pathway effectively inhibits apoptosis in the penumbra during the subacte phase after transient MCAO (23, 38). We found that most p-CREB-positive cells were colocalized with p-ERK1/2, p-p38 MAPK, and Bcl-2 in the hippocampal CA1 area. The numbers of p-CREB/p-ERK1/2- and Bcl-2/p-CREB-immunopositive cells were markedly decreased in the hippocampal CA1 area. However, 10- and 50-Hz treatments effectively restored the aforementioned proteins in the hippocampal CA1 area at 7 days after transient GCI. In addition, the 10-Hz treatment effectively up-regulated p-CREB/p-p38 MAPK expression in the hippocampal CA1 area. These findings indicate that the anti-apoptotic effects of 10- and 50-Hz treatments are most likely attributed to the activation of ERK1/2/p38 MAPK/CREB/Bcl-2-mediated signaling and ERK1/2/CREB/Bcl-2-mediated signaling, respectively, in the hippocampal CA1 area at 7 days after transient GCI.

During global cerebral I/R injury, excessive ROS production disrupts MOM integrity, resulting in the release of pro-apoptotic proteins, such as cyt c, AIF, and Smac/DIABLO, from the mitochondria into the cytosol (40). Furthermore, pro-apoptotic proteins, such as Bax and Bak, insert into the mitochondrial membrane and form homo-oligomers, which promote MOM permeabilization during ischemic stimuli (14, 41). However, Bcl-2 inhibits Bax or Bak oligomerization in the MOM and preserves mitochondrial integrity, thereby preventing the release of pro-apoptotic proteins (14, 42). The released cyt c binds to Apaf-1 and triggers the formation of an apoptosome, which activates caspase-3, the major executioner of caspase, in ischemic neurons undergoing apoptosis (43). AIF is a mammalian mitochondrial intermembrane flavoprotein. During its release from the mitochondria, AIF migrates to the nucleus and causes large-scale (~50 kbp) DNA fragmentation, leading to apoptotic neuronal death independent of caspase-3 activation (44). Smac/DIABLO released from the mitochondria can neutralize the protective effects of XIAP and subsequently promote caspase-3-mediated apoptosis after ischemic stroke (43, 45). During transient cerebral ischemia, the expression of AIF and XIAP proteins is up-regulated 1 day after reperfusion and is subsequently maintained 7 days after reperfusion (46). Up-regulation of Bcl-2 protects against apoptosis by preserving MOM integrity and inhibiting the release of cyt c, AIF, and Smac/DIABLO from the mitochondria into the cytosol in the hippocampus during the subacute phase after transient GCI (47). We found that the numbers of Bak-, Smac/DIABLO-, cyt c-, cleaved caspase-3, and AIF-immunopositive cells markedly increased in the hippocampal CA1 area, whereas the number of XIAP/NeuN-positive cells markedly decreased in the hippocampal CA1 area. However, 10- and 50-Hz treatments effectively reversed the expression of the aforementioned immunopositive cells in the hippocampal CA1 area at 7 days after transient GCI. Hence, we propose that 10- and 50-Hz treatments effectively down-regulate mitochondrial pro-apoptotic protein release into the cytosol, partly by preserving MOM integrity in the hippocampal CA1 area. Furthermore, the anti-mitochondria-related apoptotic effects of 10- and 50-Hz treatments are most likely attributed to the activation of ERK1/2/p38 MAPK/CREB/Bcl-2- and ERK1/2/CREB/Bcl-2-mediated anti-apoptotic signaling, respectively, in the hippocampal CA1 area at 7 days after transient GCI. 

**Figure 7:**
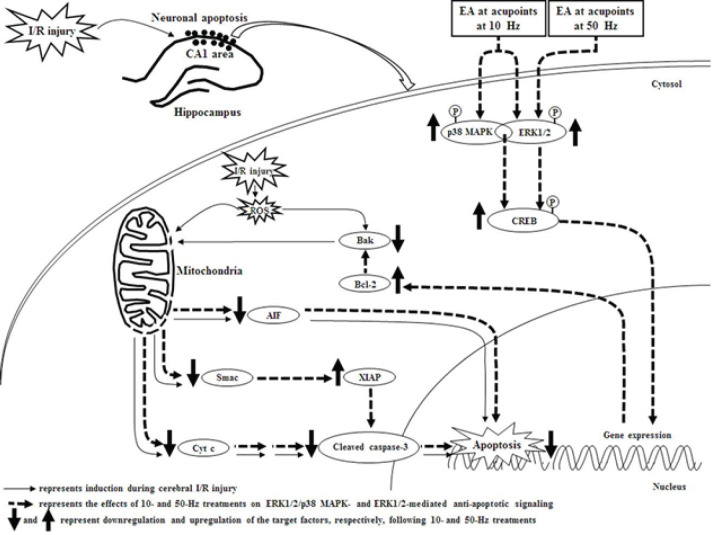
Schematic representation of the possible mechanisms of 10- and 50-Hz treatments on ERK1/2/p38 MAPK/CREB/Bcl-2- and ERK1/2/CREB/Bcl-2-mediated anti-apoptotic signaling, respectively, in the subacute phase of transient GCI

To evaluate the precise roles of ERK1/2 and p38 MAPK in the neuroprotective effects of 10- and 50-Hz treatments on mitochondria-mediated apoptotic signaling in the subacute phase after transient GCI, the rats in the D+10 Hz, D+50 Hz, U+10 Hz, U+50 Hz, and SB+10 Hz groups were ICV injected with 1% DMSO, 1% DMSO, U0126, U0126, and SB203580, respectively. Studies have shown that pretreatment with U0126 and SB203580 could effectively down-regulate ERK1/2- and p38 MAPK-mediated anti-cyt c/cleaved caspase-3 apoptotic signaling, respectively, in the ischemic areas during the subacute phases after cerebral ischemia (21, 48). We found that U0126 treatment (in the U+10 Hz and U+50 Hz groups) significantly abrogated memory improvement and the neuroprotective effects of 10- and 50-Hz treatments, including ERK1/2/p38 MAPK/CREB/Bcl-2- and ERK1/2/CREB/Bcl-2-mediated anti-cyt c/cleaved caspase-3 and AIF apoptotic signaling, respectively, in the hippocampal CA1 area. Moreover, SB203580 treatment (in the SB+10 Hz group) significantly abrogated memory improvement and the neuroprotective effects of 10-Hz treatment on the aforementioned protein expression levels. However, the effects of D+10-Hz and D+50-Hz treatments on the aforementioned apoptotic pathways in the hippocampus were similar to those of 10-Hz and 50-Hz treatments, respectively. Based on these results, we infer that 10-Hz treatment exerts neuroprotective effects against mitochondria-related apoptosis by the upstream interactions between ERK1/2 and p38 MAPK, which subsequently activate downstream CREB/Bcl-2-mediated signaling in the hippocampal CA1 area. We further propose that the neuroprotective effects of 10- and 50-Hz treatments on cyt c/cleaved caspase-3- and AIF-mediated apoptosis are attributed to the activation of ERK1/2/p38 MAPK/CREB/Bcl-2- and ERK1/2/CREB/Bcl-2-mediated signaling, respectively, in the hippocampal CA1 area at 7 days after transient GCI (Figure 7). 

**Table 1:** Primary antibodies used in this study for Western blot analysis, immunofluorescence staining, or immunohistochemistry staining

Host	Primary antibody	WB (dilution)	IF (dilution)	IHC (dilution)	Source/catalog No.
Rabbit	p-ERK1/2	1:1000			CST/#4376
Rabbit	ERK1/2	1:1000			CST/#9102
Rabbit	p-p38 MAPK	1:1000			CST/#9211
Rabbit	p38 MAPK	1:1000			CST/#9212
Rabbit	p-JNK	1:1000			CST/#9251
Rabbit	JNK	1:1000			CST/#9252
Rabbit	p-Akt	1:1000			CST/#9271
Rabbit	Akt	1:1000			CST/#4685
Mouse	Actin (loading control)	1:5000			NB/NB600-501
Rabbit	p-ERK1/2		1:100		CST/#4370
Mouse	p-CREB		1:100		Millipore/05-807
Rabbit	p-p38 MAPK		1:100		CST/#9211
Rabbit	Bcl-2		1:100		abcam/ab196495
Mouse	NeuN		1:100		Merck Millipore/MAB377
Rabbit	XIAP		1:100		Abcam/ab227196
Rabbit	Bak			1:200	CST/#12105T
Mouse	cyt c			1:50	BioVision/257-100
Rabbit	cleaved caspase-3			1:100	CST/#9664
Rabbit	Smac/DIABLO			1:50	Abcam/ab32023
Rabbit	AIF			1:50	CST/#4642

WB: Western blotting; IF: Immunofluorescence; IHC: Immunohistochemistry; CST: Cell Signaling Technology; NB: Novus Biologicals

Representative images show (A) TUNEL-, (B) p-ERK1/2 (green)/NeuN (red)-, and (C) p-p38 MAPK (green)/NeuN (red)-positive cells in the hippocampal CA1 area in the Sham, Model, Non-acup, 10 Hz, 30 Hz, 50 Hz, U+10 Hz, U+50 Hz, and SB+10 Hz groups. (D) Representative image shows a brain section of the hippocampus. The rectangle region indicates the selected CA1 area in which the numbers of immunopositive cells were calculated. The bar graphs show the numbers of (E) TUNEL- (F) p-ERK1/2/NeuN-, and (G) p-p38 MAPK/NeuN-positive cells in the hippocampal CA1 area in the experimental groups. (H) The STL of the experimental groups was evaluated at 7 days after transient GCI. STL, step-through latency. Data are presented as mean±SD. *P<0.05 versus the Sham group; #*P*<0.05 versus the Model group. Arrows in (A), (B), and (C) point to TUNEL-, p-ERK1/2/NeuN (yellow)-, and p-p38 MAPK/NeuN (Yellow)-positive cells, respectively. Scale bars in (A), (C), and (D) represent 60 μm, 50 μm, and 500 μm, respectively

(A) Representative Western blot images show p-ERK1/2, ERK1/2, p-p38 MAPK, p38 MAPK, p-JNK, JNK, p-Akt, and Akt expression in the hippocampus in the Sham, Model, Non-acup, 10 Hz, 30 Hz, and 50 Hz groups. The bar graphs show the ratios of (B) p-ERK1/2/ERK1/2, (C) p-p38 MAPK/p38 MAPK, (D) p-JNK/JNK, and (E) p-Akt/Akt expression in the hippocampus among the experimental groups. Actin is used as an internal control in western blot analysis. *P<0.05 versus the Sham group; #*P*<0.05 versus the Model group.

(A) Representative western blot images show p-ERK1/2, ERK1/2, p-p38 MAPK, and p38 MAPK expression in the hippocampus in the D+Sham, D+Model, D+10 Hz, D+50 Hz, U+10 Hz, U+50 Hz, and SB+10 Hz groups. The bar graphs show the ratios of (B) p-ERK1/2/ERK1/2 and (C) p-p38 MAPK/p38 MAPK expression in the hippocampus among the experimental groups. *P<0.05 versus the D+Sham group; #*P*<0.05 versus the D+Model group.

Representative images show the expression of (A) p-CREB (red)/p-ERK1/2 (green)-, (B) p-CREB (red)/p-p38 MAPK (green), and (C) Bcl-2 (green)/p-CREB (red)-positive cells in the hippocampal CA1 area in the Sham, Model, Non-acup, 10 Hz, 30 Hz, 50 Hz, U+10 Hz, U+50 Hz, and SB+10 Hz groups. The bar graphs show the numbers of (D) p-CREB/p-ERK1/2-, (E) p-CREB/p-p38 MAPK, and (F) Bcl-2/p-CREB-positive cells in the hippocampal CA1 area among the experimental groups. *P<0.05 versus the Sham group; #*P*<0.05 versus the Model group. Arrows in (A), (B), and (C) point to p-CREB/p-ERK1/2-, p-CREB/p-p38 MAPK, and Bcl-2/p-CREB-positive cells (yellow), respectively. Scale bar in (C) represents 50 μm.

Representative images show the expression of (A) Bak-, (B) Smac/DIABLO-, and (C) cyt c-positive cells in the hippocampal CA1 area in the Sham, Model, Non-acup, 10 Hz, 30 Hz, 50 Hz, U+10 Hz, U+50 Hz, and SB+10 Hz groups. (D) Representative image shows the negative control for Bak, Smac/DIABLO, or cyt c antibody. The bar graphs show the numbers of (E) Bak-, (F) Smac/DIABLO-, and (G) cyt c-positive cells in the hippocampal CA1 area among the experimental groups. *P<0.05 versus the Sham group; #*P*<0.05 versus the Model group. Arrows in (A), (B), and (C) point to Bak-, Smac/DIABLO-, and cyt c-positive cells, respectively. Scale bars in (C) and (D) both represent 60 μm.

Representative images show the expression of (A) XIAP (green)/NeuN (red)-, (B) cleaved caspase-3-, and (C) AIF-positive cells in the hippocampal CA1 area in the Sham, Model, Non-acup, 10 Hz, 30 Hz, 50 Hz, U+10 Hz, U+50 Hz, and SB+10 Hz groups. (D) Representative image shows the negative control for cleaved caspase-3 or AIF antibody. The bar graphs show the numbers of (E) XIAP/NeuN-, (F) cleaved caspase-3, and (G) AIF-positive cells in the hippocampal CA1 area among the experimental groups. *P<0.05 versus the Sham group; #*P*<0.05 versus the Model group. Arrows in (A), (B), and (C) point to XIAP/NeuN (yellow)-, cleaved caspase-3-, and AIF-positive cells, respectively. Scale bars in (A), (C), and (D) represent 50 μm, 60 μm, and 60 μm, respectively.

P: phosphorylated; Smac: Smac/DIABLO

## Conclusion

In this study, we found that the anti-mitochondria-related apoptotic effects of 10- and 50-Hz treatments are attributed to the activation of ERK1/2/p38 MAPK/CREB/Bcl-2- and ERK1/2/CREB/Bcl-2-mediated signaling, respectively, in the hippocampal CA1 area at 7 days after transient GCI. Thus, EA at GV14 and GV20 acupoints at 10 and 50 Hz, but not at 30 Hz, is an effective therapeutic strategy for hippocampal neuronal apoptosis in the subacute phase of global cerebral I/R injury. However, further investigation is warranted to clarify the correlation between EA at acupoints at different frequencies and the role of MAPK-mediated signaling in protecting hippocampal neurons following transient GCI.

## Authors’ Contributions

YT T and CY C designed experiments. CY C performed experiments, analyzed data, and wrote the manuscript. YT T helped to draft the manuscript.

## Data Availability

The datasets used and/or analyzed during the current study are available from the corresponding author upon reasonable request.

## Conflicts of Interestt

The authors declare that there are no conflicts of interest.
